# Acute abdomen during pregnancy: appendicular deciduosis—a case report

**DOI:** 10.1093/jscr/rjab477

**Published:** 2021-10-20

**Authors:** Mossaab Ghannouchi, Karim Nacef, Mohamed Ben KHlifa, Kraim Iyed, Moez Boudokhan, Dhekra Toumi, Olfa Zoukar

**Affiliations:** Department of Surgery, Tahar Sfar Hospital, Mahdia, Tunisia; Department of Surgery, Tahar Sfar Hospital, Mahdia, Tunisia; Department of Surgery, Tahar Sfar Hospital, Mahdia, Tunisia; Department of Surgery, Tahar Sfar Hospital, Mahdia, Tunisia; Department of Surgery, Tahar Sfar Hospital, Mahdia, Tunisia; Department of Gynecology and Obstetrics, Centre de Maternité et Néonatalogie Monastir, Tunisia; Department of Gynecology and Obstetrics, Centre de Maternité et Néonatalogie Monastir, Tunisia

## Abstract

Despite the fact that it is rarely found in the appendix during pregnancy, ectopic decidua can, in some cases, cause the occlusion of the appendicular lumen by extrinsic compression due to the expansion of endometrial tissue or due to decidua polyp formation. This condition consequently leads to appendicular inflammation. We report the case of a 27-year-old primigravida woman, 32 weeks of gestation, who presented to our facility with a 2-day history of isolated right iliac fossa pain. The diagnosis of an acute appendicitis was suspected and a planned appendectomy was performed. Microscopical examination showed appendicular deciduosis.

## INTRODUCTION

The circulating progesterone levels during pregnancy stimulate endometrial decidualization, which is an essential feature for the implantation of pregnancy. It is characterized by hypertrophy of stromal tissue, increased glandular secretion and vascular proliferation.

Rarely, can this cell transformation occur outside of the endometrium, and decidual cell groups could be found in the ovaries, uterus and cervix. A rare form of ectopic decidua can be located in the appendix and cause complication with an acute appendicitis. To date, only few cases of deciduosis resulting in acute appendicitis have been reported in the literature.

## OBSERVATION

A 27-year-old primigravida woman, 32 weeks of gestation, with unremarkable medical and surgical history, presented to our facility with a 2-day history of isolated right iliac fossa pain.

No nausea, vomiting or urinary symptoms were reported and there were no similar previous episodes of pain. The body temperature was 38.4°. Abdominal palpation revealed tenderness in the right iliac fossa. The white blood cell count was 22 450/mm^3^ and the level of C-reactive protein (CRP) reached 67.7 mg/l. The urine analysis was normal. The abdominal ultrasonography confirmed a viable intrauterine pregnancy and did not show the appendix or any other abnormalities. Magnetic resonance imaging investigation was not available in our institution.

Given the suspicion for acute appendicitis, we decided to perform a planned appendectomy.

The intraoperative findings included inflamed appendix. No other peritoneal or pelvic pathologies were identified and a conventional appendectomy was performed.

A pelvic ultrasonography performed after surgery, showed normal fetal movements.

The patient was discharged on post-operative Day 2, and gave birth to a healthy boy 7 weeks later.

**Figure 1:**
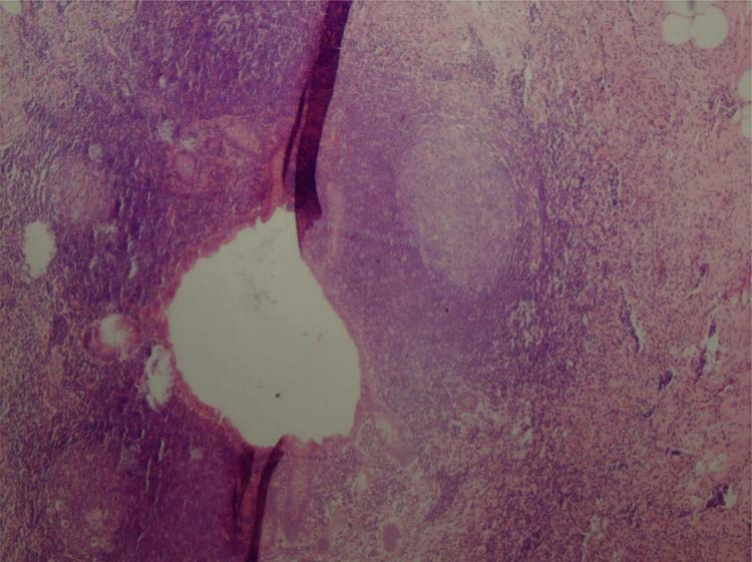
Constricted appendicular lumen.

**Figure 2:**
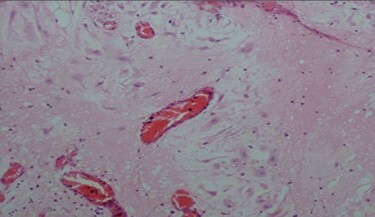
Decidual cells with abundant cytoplasm.

**Figure 3:**
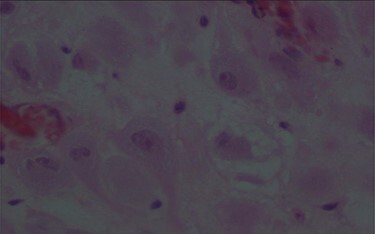
Large decidual cell with abundant eosinophilic cytoplasm and round nuclei with prominent nucleoli.

The pathologic examination showed an appendix of 6 × 1.5 cm with a constricted lumen suggesting appendiceal obstruction (Fig. 1). Microscopic examination showed subserosal deciduosis with large polygonal decidual cells, eosinophilic abundant cytoplasm and round nuclei with prominent nucleoli. A hyperplasic lymphoid reaction was found in the submucosa and mucosa (Figs 2 and 3).

## DISCUSSION

The pathogenesis of ectopic decidua and precise mechanism reactions are not yet fully understood and it is controversial to decide whether it is a physiological reaction or rather a pathological process. Deciduosis may be a de novo development of decidua originated from submesothelial stroma, or a decidual transformation of a pre-existing endometriosis [1].

Commonly, ectopic decidua is a benign condition that is not responsible for any symptoms. It regresses within 4–6 weeks in the postpartum without any treatment but it may reappear in a subsequent pregnancy [1, 2].

However, in some cases it can cause abdominal pain, intraperitoneal hemorrhage, hydronephrosis or hematuria secondary to renal or pelvis involvement, it can rarely cause right iliac fossa pain or acute appendicitis [3]. In this case, appendicular transformation caused by decidualization can be explained by two theories. The first one is extrinsic compression of the appendiceal lumen by the expansion of endometrial tissue undergoing decidualization [4]. The second one, involving intraluminal decidua polyp formation [5]. The final common pathway of both mechanisms is increased pressure with a constricted appendicular lumen [6]. It can mimic acute appendicitis in pregnancy and be responsible for abdominal pain and leukocytosis.

Deciduosis of the appendix has been rarely reported in the literature. To date only few cases have been reported [1, 4, 6–8].

Regarding the differential diagnosis with malignant tumors, a morphological examination of the removed tissues is the ‘gold standard’. Deciduosis is characterized by nodular architecture, composed of large cells with well-defined borders and eosinophilic cytoplasm [7]. Decidual cells are typically perivascular. They appear as large cells with an eosinophilic cytoplasm, and round nuclei with prominent nucleoli. Mitotic activity and/or nuclear atypia are absent. Immunohistochemistry staining is used when deciduosis presents pseudo-tumoral or infiltrative characteristics in order to exclude a peritoneal mesothelioma or a primary or metastatic peritoneal cancer [9].

Decidual cells are positive to vimentin and CD10. They also have nuclear receptors of progesterone and estrogens, but they are negative to cytokeratin and calretinin [10].

Treatment of appendicular deciduosis is surgery with standard follow up.

In conclusion, deciduosis of the appendix resulting in acute appendicitis is a rare pathology. Unlike other sites of ectopic deciduosis that can be managed expectantly, the treatment is appendicectomy. Histological examination is mandatory in order to exclude a peritoneal mesothelioma or a primary or metastatic peritoneal cancer.
